# Nutritional and Physicochemical Characteristics of Innovative High Energy and Protein Fruit- and Date-Based Bars

**DOI:** 10.3390/foods12142777

**Published:** 2023-07-21

**Authors:** Hani A. Alfheeaid, Hassan Barakat, Sami A. Althwab, Khalid Hamid Musa, Dalia Malkova

**Affiliations:** 1 Department of Food Science and Human Nutrition, College of Agriculture and Veterinary Medicine, Qassim University, Buraydah 51452, Saudi Arabia; h.alfheeaid@qu.edu.sa (H.A.A.); thaoab@qu.edu.sa (S.A.A.); k.musa@qu.edu.sa (K.H.M.); 2 School of Medicine, Dentistry and Nursing, College of Medical, Veterinary and Life Sciences, University of Glasgow, Glasgow G12 8QQ, UK; dalia.malkova@glasgow.ac.uk; 3 Food Technology Department, Faculty of Agriculture, Benha University, Moshtohor 13736, Egypt

**Keywords:** *Phoenix dactylifera*, date bars, high protein, high energy, nutrition, food supply

## Abstract

With the increasing global nutritional bar market, developing and formulating innovative high-energy and protein bars to compensate for nutrients using date fruits is beneficial for health-conscious individuals. The current research was undertaken to study the composition and physicochemical characteristics of innovative high-energy and high-protein bars using two combinations of Sukkari dates or fruit mixtures as a base. Fifty percent of either Sukkari date paste or dried fruit mixture (25% raisin, 12.5% fig, and 12.5% apricot) combined with other different ingredients was used to produce a date-based bar (DBB) or fruit-based bar (FBB). Proximate composition, sugar content, amino and fatty acid profiles, minerals and vitamins, phytochemicals, antioxidant activity, and visual color parameters of the DBB and the FBB were determined and statistically compared. Proximate analysis revealed higher moisture and fat content in the FBB than the DBB, while ash and crude fiber were higher in the DBB than the FBB. The protein content in the DBB and the FBB was not statistically different. Both prepared bars exuded around 376–378 kcal 100 g^−1^ fresh weight. Sugar profile analysis of the DBB and the FBB showed dependable changes based on date or fruit content. Fructose, glucose, and maltose contents were higher in the FBB than in the DBB, while sucrose content was higher in the DBB than in the FBB. The DBB showed significantly higher content in Ca, Cu, Fe, Zn, Mn, and Se and significantly lower content in Mg, K, and Na than the FBB, with no variation in phosphorus content. The DBB and the FBB contained both essential (EAA) and non-essential (NEAA) amino acids. The DBB scored higher Lysine, Methionine, Histidine, Threonine, Phenylalanine, Isoleucine, and Cystine contents than the FBB, while the FBB scored only higher Leucine and Valine contents than the DBB. Seventeen saturated fatty acids were identified in the DBB and the FBB, with Palmitic acid (C16:0) as the predominant fatty acid. Oleic acid (C18:1n9c) was predominant among seven determined monounsaturated fatty acids. Linoleic fatty acid (C18:2n6c) was predominant among eight identified polyunsaturated fatty acids. In addition, α-Linolenic (C18:3n3) was detected in a considerable amount. However, in both the DBB and the FBB, the content and distribution of fatty acids were not remarkably changed. Regarding phytochemicals and bioactive compounds, the FBB was significantly higher in total phenolic content (TPC), total flavonoids (TF), and total flavonols (TFL) contents and scavenging activity against DPPH and ABTS free radicals than the DBB. The DBB and the FBB showed positive a* values, indicating a reddish color. The b* values were 27.81 and 28.54 for the DBB and the FBB, respectively. The DBB is affected by the lower L* value and higher browning index (BI) to make its color brownish. Sensory evaluation data showed that panelists significantly preferred the DBB over the FBB. In conclusion, processing and comparing these bars indicated that using Sukkari dates is a nutrient-dense, convenient, economical, and better sugar alternative that helps combat the calorie content. Thus, scaling up the use of dates instead of fruits in producing high-energy and protein bars commercially is highly recommended.

## 1. Introduction

*Phoenix dactylifera* L., a member of the Arecaceae family, is a palm date tree essential to the economies and diets of the Middle East and North Africa [1]. Saudi Arabia (SA) is the second-largest producer of palm dates worldwide, supplying almost 16% of the world’s date fruit supply from growing 450 date palm cultivars [2]. Most of these products meet the universal premium quality standards; however, the quantity of low-quality fruits (10–15%) is not marketable and comprises about 150 kilo tons annually, extremely sold at low prices (almost 0.3 USD per kg) [3]. Thus, incorporating them in the processing of many food products, including bakery products and energy chocolate bars, will be an excellent choice. Amazingly, various studies have documented the nutritional value of common date fruits [1,2,4,5]. Dates are the primary source for creating date pastes and date syrup, which are used in the confectionery and fermentation industries [6]. It is also a good source of vitamins A and C, as well as B vitamins like thiamine and riboflavin [1,7], and studies have shown that dates induce antioxidant [8,9], anti-cancer [10], and antiviral [11] activities. Date fruits have a relatively high percentage of carbohydrates (71.2–81.4% of total energy content) and ash (1.68–3.94%) relative to their fat and protein content (0.12–0.72% and 1.72–4.73%, respectively) [5,12]. The edible flesh of dates is a valuable and available source of mainly fructose, glucose, sucrose, 5–8.5% dietary fiber, and considerable amounts of polyphenols [13,14]. Sukkari, Barhi, Shagraa, Wanana, Khalas, Hushana, Maktoomi, Khadra, Mitwah, Slamiya, Rushodia, Ruthana, Sabaka, Segae, Mabroom, Mutwah, Meneifi, Hulwa, Nabtat Ali, Nabtat Rashid, Aseela, and Khodry are the most popular varieties of date fruits [3]. Sukkari dates contain approximately 78% CHO, 2.8% protein, 2.5% fats, 5% fiber, and 2.3% minerals, providing high caloric value [15]. The predominant mineral is potassium, and its protein structure includes many essential amino acids [5]. Moreover, dates contain carotenoids [16] and phytochemicals [17], providing valuable nutritional and therapeutic properties.

Date-based bars (DBBs) are preferable to fresh dates because they are a more convenient and portable source of balanced and dense nutrients, especially out of season. DBBs can also address the growing demand for this fruit from local and international consumers [18]. A healthy lifestyle encourages people to regularly eat more fruit, including nutritious fruit bars successfully developed in recent years from different fruits [2,19]. Available fruit-based bars deliver superior nutritional and energy values and appear to be exceptional instant foods, providing the necessary macro- and micro-nutrients, dietary fibers, and bioactive compounds [20]. A few studies confirmed that producing different date-based bars could also be valuable and applicable [2,21,22,23,24]. Research findings suggested that DBBs could be necessary to meet health-conscious customer requirements [25] and might be anticipated to achieve greater international marketability [2,25]. Multiple date-based bar manufacturing experiments have been conducted internationally. Kamel and Kramer [26] used skim milk powder and walnuts to make a simple date bar recipe. Similarly, Khalil and co-researchers [27] formulated bars using soy protein isolate, skim milk powder, and yeast proteins. Milk powder was used in several later trials because of its promising food industry implications [28]. Adding 7–11% oat flakes, 6% sesame seeds, and almonds to skim milk powder has been investigated [29].

Interestingly, Al-Hooti et al. [29] tested the mineral content of four different types of dates and found that their types independently varied. Nabtat-Ali and Sukkari, two other date varieties, were used in a separate study and had distinct impacts on proximate composition [2]. Date bars were formulated using skim milk powder, soybean flour, or almond flour [30]. The dates used by Irshad et al. [30] had a high vitamin C concentration in the immature phases of the dates, but this decreased dramatically as the dates matured [31]. The intense flavor that nuts impart due to their nutritional density makes them a fantastic addition to bars [32]. Oats, chickpeas, and soy flour were also incorporated [33]. Munir et al. [22] made date bars with roasted oatmeal flour, chickpea flour, skim milk powder, almonds, pistachios, cardamom, and carboxy methyl cellulose. Roasted chickpea flour, rice flour, and dried apricots were tested with cheddar cheese and whey protein isolate [34,35]. Bioactive components came from dates and apricots, while cheddar cheese and whey protein provided protein. Finding a good protein source that does not ruin the bars’ taste is demanding. The lowest sensory quality was seen in bars enriched with sunflower protein concentrate and isolate [36]. Another study employed whey protein concentrate and protein isolate, increasing protein content and taste [37]. Supplements boost the nutritional value of date bars. A recent study reinforced Sukkari date bars with germinated flax seed powder [38]. The amount of protein, fat, and minerals was significantly increased with this approach [39,40]. However, date bars have been made from date fruit waste in recent years [41]. Date pit powder was utilized in developing date bars that also included soy protein isolate. According to a recent summary, there is a growing interest in making date bars at home using a wide variety of recipes and components [42].

The trials above suggested that making ideal and balanced date bars with nutrients may be challenging. Since dates are not a protein source, conventional formulations focused on protein enrichment to make balanced, protein-rich bars are demanding. Also, sensory qualities are crucial to reflecting acceptance and palatability. Thus, creating a DBB may better improve health outcomes than other nutritional bars made from fruits other than dates. Therefore, this study aimed to produce a novel DBB using Sukkari dates and fruit-based bars (FBB) utilizing a mix of dried grapes, figs, and apricots. Proximate composition, sugar profile, mineral and vitamin content, amino and fatty acid profiles, phytochemicals, antioxidant activity, and visual color parameters of DBBs and FBBs were investigated, and then the data were interpreted comparatively.

## 2. Materials and Methods

### 2.1. Ingredients

Ingredients such as Sukari dates paste were purchased from Al Emtyaz Dates Factory (https://www.alemtyaz.com.sa (accessed on 2 April 2022). Dried apricot, dried fig, raisin, Sukari dates syrup, glucose syrup, sesame seeds, walnuts, whole oat, coconut powder, cow’s samna, peanut butter, and edible salt were obtained from Al-Tamimi market (https://www.tamimimarkets.com (accessed on 3 April 2022) in Al Qassim region, Saudi Arabia. Oat fiber (NuNaturals, Eugene, OR, USA) was ordered online from iHerb (https://sa.iherb.com (accessed on 24 March 2022), while milk protein concentrate and whey protein isolate were purchased from MYPROTEIN Co., Manchester, UK (www.myprotein.com (accessed on 15 March 2022).

### 2.2. Formulation of Date-Based and Fruit-Based Bars

The procedure of Ibrahim et al. [24] has been modified depending on the formulas in Table 1. First, walnuts and whole oats were lightly ground (Severin, type Km 3871, Mecklenburg, Germany) at speed 3 for 30 s. Next, dried ingredients such as sesame seeds, ground walnuts, whole oat powder, and oat fiber were added, while milk protein concentrate, whey protein isolate, and salt were roasted with continuous stirring in an air-heated oven at 200 °C for 5 min, then coconut powder was added. The whole mix was stirred for over 2 min. For the DBB, the other ingredients, such as date paste, cow’s samna, dates syrup, and peanut butter, were warmed in an air-heated oven at 120 °C for 5 min to prepare the date mix. For the Fruit-based bar (FBB), dried apricot, dried fig, and raisin were live steamed for 5 min, then mixed with cow’s samna, glucose syrup, and peanut butter, and then warmed in an air-heated oven at 120 °C for 5 min, then homogenized using a knife blender (Santos, VITA-MAX CORP-Light Industrial Food Preparing Machine Model, VM0122E, Cleveland, OH, USA) at speed 4 for 2 min to prepare the fruit mix before further processing. Next, the roasted dried mixture was mixed with the date or fruit mix in a dough mixer until a homogenized paste was obtained. Finally, the DBB and the FBB were weighed and shaped using a ten-hole frame to get both bars. The prepared bars were then cooled down to 4 °C in the refrigerator until further analysis or utilization.

### 2.3. Proximate Chemical Composition, Minerals, and Vitamins Content in DBB and FBB

The formulated DBB and FBB were subjected to chemical analysis (moisture, crude protein, crude lipids, ash, dietary fibers, and available carbohydrates) and caloric values according to the methods of A.O.A.C. [43]. The content of minerals, including sodium and potassium, was determined using flame photometry. Comparatively, atomic absorption spectroscopy was used to determine the concentrations of Ca, Mg, Fe, Cu, Mn, and Zn using the protocol of A.O.A.C. [43]. In addition, a standard colorimetric method was employed for phosphorus, as mentioned by Borah et al. [44]. Vitamin content was individually determined using HPLC analysis [45].

### 2.4. Phytochemical Analysis of DBB and FBB

Total phenolic compounds (TPC) in the DBB and the FBB were determined using the Folin-Ciocalteau method, and TPC was expressed as milligram Gallic acid equivalents (mg GAE 100 g^−1^ dw) according to Bettaieb et al. [46]. Total carotenoids (TC) content was determined colorimetrically as described in the modified method [47]. The antioxidant activity as DPPH radical scavenging activity (DPPH-RSA) was determined colorimetrically using 2,2-diphenylpicrylhy-drazyl (DPPH) radicals. The DPPH free radical inhibition percentage was calculated; the results were interpreted toward plotting the Trolox standard and presented as μmol TE g^−1^ dw [48]. Total flavonoid (TF) and total flavonols (TFL) contents have been determined, and the results were presented as mg quercetin equivalent (QE) g^−1^ using the methods of Barakat and Almundarij [49] and Kumaran and Karunakaran [50], respectively.

### 2.5. Determination of the Amino Acid Profile of DBB and FBB

The amino acid profiles of the DBB and the FBB were determined using HPLC-PICO-TAG upon hydrolysis under acidic conditions in evacuated ampoules at 110 °C for 24 h. Quantitative determination of amino acids was carried out according to Cohen et al. [51]. According to Blouth et al. [52], tryptophan was colorimetrically measured in the alkaline hydrolysate. The predicted biological value (BV) [53] and amino acid score [54] were estimated in vitro.

### 2.6. Determination of the Fatty Acid Profile of DBB and FBB

According to Aldai et al. [55], total fatty acid fractions were methylated. The methyl esters of fatty acids (FAs) obtained from the DBB and the FBB were determined using a GLC equipped with a flame ionization detector (FID). The GLC conditions were updated using an incremental elevated temperature program from 100 to 200 °C at different times, followed by 10 min at 2 °C min^−1^ to 230 °C, then held for 10 min. The injection temperature was 250 °C. The detector temperature was 300 °C. Results were evaluated with a conventional integrator program (Saturn GC Workstation Software ver., 5.51).

### 2.7. Instrumental Color Measurements of DBB and FBB

The color of each sample was measured via a Chromameter (ColorFlex, Reston, AV, USA) applying the CIELAB scale (L*, a*, and b*) adjusted with typical white, green, and black tiles. The hue angle (Hᵒ), chroma (C), and browning index (BI) were then calculated according to Lavelli et al. [56] using the continuity equations (Equations (1)–(3)):

(1) $$C=(a^{2}+b^{2}{)}^{0.5}$$

(2) $$H^{{}^{\circ}}=\tan^{-1}(\frac{b}{a})$$

(3) $$\mathrm{BI}=\frac{[100\left(\frac{\left(a+1.75L\right)}{\left(5.645L+a\;-\;3.012b\right)}\right)-\;0.31]}{0.17}$$

### 2.8. Sensory Evaluation

According to Gámbaro and McSweeney [57], a 9-hedonic scale ranging from 1 = Dislike extremely to 9 = Like extremely was used to evaluate the formulated bars organoleptically. Freshly manufactured DBBs and FBBs were given to 12 trained panelists. Attributes such as appearance, taste, color, odor, texture, mouthfeel, and overall acceptability were examined. Sensory tests were conducted at the Department of Food Sciences and Human Nutrition, College of Agriculture and Veterinary Medicine, Qassim University, KSA, in a prepared lab under 21 ± 1 °C and potable water was provided for each panelist as a taste purifier before tasting each bar.

### 2.9. Statistical Analysis

The statistical analysis was carried out using a one-way analysis of variance (ANOVA) using SPSS, ver. 22 (IBM Corp. Armonk, NY, USA, Released 2013). Data were treated as a complete randomization design, multiple comparisons were carried out by applying the Duncan test, and the significance level was 0.05 [58]; analysis was performed in triplicates.

## 3. Results

### 3.1. Proximate Composition of Formulated DBB and FBB

The results of the proximate chemical composition of the formulated DBB and FBB are shown in Table 2. The moisture content in the FBB was significantly higher than in the DBB, representing 13.14 and 11.22%, respectively. The protein content did not differ considerably between the DBB and the FBB, while the fat content was lower and the total CHO content was higher in the DBB. Crude fiber and ash contents were significantly higher in the DBB than in the FBB. In the present research, developed high-energy, high-protein bars have excellent protein, carbohydrate, fat, and fiber contents and could provide 378 and 376 kcal 100 g^−1^ for DBBs and FBBs, respectively.

### 3.2. Sugar Profile of Formulated DBB and FBB

HPLC determined the amount of sugars in the DBB and the FBB; the results are presented in Table 3. It could be noticed that the FBB showed higher fructose, glucose, and maltose contents, while the DBB was significantly higher in sucrose content only. Obviously, the formulated DBB could provide low glucose and fructose, which could have a low glycemic response after consumption. Lactose was lower than the detection limit in both prepared bars.

### 3.3. Mineral and Vitamin Contents of Formulated DBB and FBB

The formulated DBB and FBB were analyzed for their mineral contents. Using different base materials in both bars may be the reason behind the different ratios of minerals in the presented bars (Table 4). However, the DBB showed significantly higher content in Ca, Cu, Fe, Zn, Mn, and Se than the FBB. On the contrary, the FBB exhibited higher content in Mg, K, and Na than the DBB. In comparison, approximately equal phosphorus was observed in both the DBB and the FBB.

In the same context, the vitamin content of the DBB and the FBB is presented in Table 5. Interestingly, the DBB showed a higher content of vitamin D_3_, vitamin E, vitamin B_2_, vitamin B_5_, and vitamin B_6_. On the contrary, the FBB was significantly higher in vitamin A and B_12_ than the DBB. However, both bars presented vitamin C, B_1_, and B_3_ in traces.

### 3.4. Amino Acid Composition of Formulated DBB and FBB

The DBB and FBB were analyzed for their contents of amino acids, and the data are shown in Table 6. Both samples showed a good and balanced profile of all amino acids analyzed, reflecting their nutritional values. Most essential and non-essential amino acids were present in both the DBB and the FBB. The DBB scored higher individual essential amino acids (EAA) in seven amino acids (Lysine, Methionine, Histidine, Threonine, Phenylalanine, Isoleucine, and Cystine) than the FBB, while the FBB scored only higher individual essential amino acids in two amino acids (Leucine and Valine) than the DBB. However, the total EAA content in the DBB was slightly higher in the FBB, representing 8.27 and 8.13 g 100 g^−1^ bar, respectively. Regarding the non-essential amino acids (NEAA), the DBB showed higher content in Aspartic, Glutamic, Arginine, Alanine, and Tyrosine than the FBB. Serine, Glycine, and Proline contents presented higher content in the FBB than in the DBB. In the same context, the FBB showed a higher NEAA than the DBB. Interestingly, the EAA/NEAA ratio was higher in the DBB than in the FBB.

Table 7 displays the amino acid%, computed biological efficiency, essential amino acid index (EAAI), and requirement index of different prepared bars. Total basic amino acid (BAAs) content, such as Lysine, Arginine, and Histidine, increased in the DBB more than in the FBB. An Opposite finding was found in the FBB than the DBB regarding total uncharged polar AAs. The calculated BV and EAAI values increased in the DBB more than in the FBB. However, both prepared bars are preferred and served to various age groups; the requirement index depending on the World health organization was calculated, and the results are presented in Table 7. Naturally, adding the Sukkari date instead of the fruit mix raised the nutritional value and requirement index in all age groups.

### 3.5. Fatty Acid Composition of Formulated DBB and FBB

The results of the fatty acid composition in the DBB and the FBB are shown in Table 8. Palmitic acid (C16:0) was the most abundant saturated fatty acid in formulated bars, followed by Myristic (C14:0) and Stearic (C18:0) acids. The total saturated fatty acids were presented as 34.35 and 33.98 g 100 g^−1^ fat for the DBB and the FBB, respectively. For the monounsaturated fatty acids, 27.3 and 27.9 g 100 g^−1^ fat were recorded for the DBB and the FBB, respectively. The most abundant monounsaturated fatty acid was oleic acid (C18:1n-9c), representing 26.2 and 26.5 g 100 g^−1^ fat in the DBB and the FBB, respectively. Linoleic (C18:2n-6c), as a polyunsaturated fatty acid, was 31.70 and 32.11 g 100 g^−1^ fat in the DBB and the FBB, respectively. Also, α-Linolenic (C18:3n-3) was detected by 5.97 and 5.87 in the DBB and the FBB, respectively.

### 3.6. Antioxidant Activities

Total phenolic content (TPC), total flavonoid (TF), total flavonols (TFL), 2,2-diphenyl-1-picrylhydrazyl radical scavenging activity (DPPH-RSA), and 2,2′-Azino-bis(3-ethylbenzthiazoline-6-sulfonic acid) radical scavenging activity (ABTS-RSA) were determined in the formulated DBB and FBB, and results are presented in Table 9. The FBB was significantly higher in TPC, TF, and TFL contents and scavenging activity against DPPH and ABTS free radicals than the DBB.

### 3.7. Visual Instrumental Color of DBB and FBB

Table 10 displays the findings of the DBB and the FBB color measurements. When comparing the FBB and the DBB, the L* value (a measure of brightness) was significantly (*p* < 0.05) higher in the FBB than in the DBB. In addition, the mean a* value (a measure of how red or green a color is) was substantially (*p* < 0.05) greater in the FBB than in the DBB. The b* value (which indicates how much of a yellow hue an object has) and the C value, on the other hand, were not significantly different. The H° and BI presented a significant difference between the DBB and the FBB. The Browning index (BI) illustrated the considerable change in visual color using date paste vs. fruit mix.

### 3.8. Sensory Evaluation

Table 11 illustrates the sensory evaluation of the formulated DBB and FBB. Results indicated that appearance and odor were not significantly affected. In contrast, significant differences were found between the DBB and the FBB in color, taste, texture, and mouthfeel. In addition, overall acceptability data showed that panelists significantly preferred the DBB over the FBB.

## 4. Discussion

The global snack bar industry is predicted to expand from 15 billion US dollars in 2019 to 19 billion by 2025 [59]. There are hundreds of on-the-go snack bars, including balanced, protein-rich cereal breakfast substitutes and brain-boosting [35]. Snack bars usually contain cereals, legumes, fruits, nuts, and chocolate chips [35]. Fruit-based snack bars are the healthiest, with natural sugars, vitamins, minerals, and other bio-nutrients to fulfill consumers’ daily nutritional needs [25]. Snack bars can benefit from the date palm fruit’s excellent nutritional content. Dates are a good source of good-quality nutrients such as dietary fiber, unsaturated fatty acids, and a wide range of micronutrients. Dates are low in protein but high in lysine and histidine. Bioactive phytochemicals such as phenolic acids, polyphenols, and carotenoids were abundant in date fruits [5,60,61,62]. Dates have functional or pharmacological benefits, according to recent in vivo and in vitro studies [42]. Dates have anti-inflammatory, anti-tumor, antihypertensive, anti-hypercholesterolemia, and antimicrobial properties [63,64,65,66,67,68,69]. Thus, our current research examined the Sukkari date fruit’s potential use in high-energy and protein-rich date-based bars. The nutritional and physicochemical properties of formulated FBBs were compared with those of formulated DBBs.

Proximate chemical analytical data concluded that FBBs and DBBs, which were typically manufactured, presented higher moisture and fat content in FBB than DBB while presenting higher ash and crude fiber in DBBs than FBBs, whereas protein content was not significantly changed. However, both prepared bars are nutrient-dense, high-energy, and high-protein, providing around 376–378 kcal 100 g^−1^ fresh weight. The increased moisture content in FBBs may be due to the manufacturing wetting process, which increased the moisture content. The fat content of dates and fruits is extremely low, specifically less than 1% of the whole fruit. Three cultivars, such as Sukkari, Burni, and Labanah, had a maximum fat content of 0.5 to 0.7%, while Khodari and Mabroom had a minimum of 0.1 to 0.2% [5,70]. Dates vary in nutritional content depending on the fruiting stage; however, fresh dates have 0.1−0.2% fat and dried dates have 0.1−0.5% [71]. Also, fruits are a poor source of fats. As remarked in the FBB, fat content was higher than that of the DBB, which may be due to added oils such as paraffin oil during the manufacturing of dried fruits. Oils are commercially used as a coating or releasing agent during the drying of fruits [72], which may release residues and increase the fat content in the FBB more than in the DBB. Fiber content was significantly higher in the DBB than in the FBB, with an average amount of 9 and 8 g 100 g^−1^ in the DBB and the FBB, respectively. The recommended daily intake of dietary fiber is 25–35 g daily for adults (25–32 for women and 30–35 g for men). It is difficult to achieve such an amount in Western societies, as they are much lower than these recommendations [73]. Even a slightly higher amount of fiber in the DBB might facilitate meeting recommendations for fiber intake. The benefits of dietary fiber intake relate to its ability to slow gastric emptying and the macronutrient digestion and absorption rate [74]. In addition, it might enhance satiety and better appetite regulation, enabling body weight reduction [75]. Not less importantly, a higher provision of fiber in the DBB would be expected to favorably impact cardiometabolic risk factors since dietary fiber effectively lowers total and low-density lipoproteins and reduces postprandial glucose concentration [76,77]. It is also essential to appreciate that the health-related benefits of dietary fiber present in DBBs and FBBs might differ due to the difference in fiber types and their physicochemical properties, such as water solubility, viscosity, and fermentability [73]. In addition, diminished fiber intake may contribute to a reported increase in the concentration of the hunger hormone ghrelin and a reduction in gastrointestinal satiety hormones, including GLP-1, thus compromising body weight maintenance [78].

Sugar profile analysis of DBBs and FBBs showed dependable changes based on date or fruit content. Fructose, glucose, and maltose presented higher content in FBBs than DBBs because of the fruit mix and added glucose syrup. In comparison, sucrose content was higher in DBBs than in FBBs because Sukkari dates contain higher sucrose content and added date syrup. Obviously, adding glucose syrup during manufacturing increased the glucose content in FBBs more than in DBBs. However, evaluated sugars in dates such as glucose, fructose, and sucrose varied in amounts ranging from 2 to 95% depending on the dates’ variety; the highest glucose content ranged from 49.6 to 95.4% in the Khalas variety. Fructose content was lower than glucose, whereas sucrose ranged from 17% to 31% [62]. Increased sugar consumption is essential to the worldwide epidemics of obesity and diabetes and their associated cardiometabolic risks [79]. It is important to note that the sugar profiles of DBBs and FBBs have several differences, with DBBs having a lower fructose and glucose content at the expense of a higher sucrose content than FBBs. Fructose is known to be a particularly harmful sugar [80], with high habitual fructose intake facilitating the development of central features of the metabolic syndrome. Indeed, enhanced fructose consumption leads to insulin resistance, promotes lipid accumulation in the liver, and is detrimental to the metabolism of TAG-rich lipoproteins, leading to hypertriglyceridemia [81]. Thus, a lower fructose content in DBBs might be expected to make this bar ‘healthier’ compared to FBBs. Consumption of DBBs might also have a less detrimental impact on cardiometabolic risk factors and insulin resistance due to their lower glucose content. This sugar produces the most rapid increase in postprandial blood glucose concentration [82]. These results indicate significant variations between dates depending on the variety and geographical conditions. Depending on geographical conditions, other date types like Ajwa have glucose, fructose, and sucrose levels of 35 to 54.5, 39 to 52.5, and 0 to 13.4%, respectively. The diverse analytical procedures used by researchers can significantly affect the obtained data, making quantitative comparisons between date varieties meaningless. However, date fruits are generally considered rich in carbohydrates [60]. Dates are high in sugar, yet El Abed et al. [83] found that dates’ aqueous extract at 200 mg kg^−1^ BW reduced postprandial glycemia in animal models. These effects were connected to inhibitory activity against type 2 diabetes-related enzymes such as α-glucosidase, which regulates intestinal glucose absorption [84]. Diabetes patients who ate different date types had decreased glycemia, according to a meta-analysis in the current systematic review [85]. In a randomized controlled trial of type 2 diabetics, 50 g of Lulu dates given daily with regular oral antidiabetic medications for two weeks did not influence blood glucose levels [86].

Formulated DBBs showed higher mineral content than FBBs, which might result from the high mineral content of date fruits. Compared to pomegranate and mango, Sukkari dates had more minerals [70]. Others claimed that some dates have 2.5 times more potassium than bananas [71]. This may be a reasonable explanation for why DBBs have a higher mineral content than FBBs. As shown in Table 1, 50% of the formulated bars are Sukkari date paste or wetted fruit mix, which could provide a high carbohydrate content. Dates are generally considered to be rich in carbohydrates [60]. Several studies have examined various date types. Burni had the most total carbs at over 80%, while Khasab had the lowest at less than 50% [60]. Like Burin, the total carbohydrate content of the date varieties Ajwa, Khodari, Labanah, and Sukkari is relatively high, ranging from 71% to 79% [5]. Dates are high in dietary fiber, ranging from 2 to 8%. Saudi Arabian Sukkari dates have a median fiber content of 4.35% [87]. Dates are an excellent source of fiber, better than cereals and fruits, because they include high-quality fiber fractions like β-glucans, arabinoxylans, and cellulose [60]. Also, dates provide 1.7–4.7% protein, which is more than other fruits [5,25,71]. Date cultivars such as Khalas, Sukkari, Barhi, Lulu, Deglet-Noor, and Medjool had median protein levels of 2−3% [60]. Protein concentration varies based on the date’s fruiting state, with fresh dates containing 1.1 to 2.0% while dried dates contain 1.5 to 3.0% [71].

In the same context, DBBs showed significantly higher content of vitamin D_3_, vitamin E, vitamin B_2_, vitamin B_5_, and vitamin B_6_. On the contrary, FBBs were considerably higher in vitamin A and B_12_ than DBBs. However, both bars presented vitamin C, B_1_, and B_3_ in traces. Dates are rich in nutrients, especially the B vitamins; vitamins B_1_, B_2_, and B_3_ were detected in several date varietals at concentrations of 0.050–0.66, 0.060–0.66, and 1.27–1.61 mg 100 g^−1^, respectively [61]. Other investigations discovered reasonably high levels of tocopherols in the form of α-, β-, and γ-tocopherol at 0.07−0.21, 0.01−0.03, and 0.01−0.04 ng 100 g^−1^ based on fresh weight, respectively [62]. Indeed, vitamin depletion during drying causes dates’ vitamin content to vary significantly between the fresh and dry phases [25,71].

DBBs and FBBs showed an excellent profile of all amino acids analyzed, reflecting their nutritional values. Most essential and non-essential amino acids were present in both DBBs and FBBs. The DBBs scored higher individual essential amino acids (EAA) such as Lysine, Methionine, Histidine, Threonine, Phenylalanine, Isoleucine, and Cystine than the FBBs, while the FBBs scored only higher Leucine and Valine than the DBBs. Our results were strongly confirmed by Amadou [61], who claimed that dates have a healthy balance of essential and non-essential acids, including proline, lysine, histidine, tyrosine, isoleucine, and tryptophan in their amino acid profile. Lysin, an essential amino acid missing in most cereals, has been detected in dates in quantities ranging from 0.025 to 0.073%, with Ajwa dates having the highest concentration. Some of the acids presented may also be lacking in common fruits like oranges, bananas, and apples. Dates have 800 times more isoleucine than apples, while lysin is 2000 to 5000 times greater than apples, bananas, and oranges [61]. Indeed, formulating DBBs with the Sukkari date slightly increased the EAA content in DBBs more than in FBBs. However, the EAA/NEAA ratio was higher in the DBBs than in the FBBs.

Interestingly, the DBB’s BV, EAAI, and requirement index changed more than those of the FBB’s. DBBs have a higher BAA content than FBBs, especially in Lysine, Arginine, and Histidine. Basic amino acids increase protein bioactivity significantly [88] and have antioxidant and antibacterial properties [89]. In contrast, total uncharged polar AAs (Glycine, Serine, Threonine, Tyrosine, and Cystine) have shown an increase in FBBs than DBBs. Singh and Sogi [90] stated that uncharged polar AAs increase protein solubility. Due to DBB’s high EAA composition, utilizing Sukkari dates instead of fruit mix also increased calculated BV and EAAI. According to amino acid requirements [54], substituting Sukkari dates for fruit mix increased nutritional value. It continuously improved the DBBs’ protein content to meet different age groups’ needs.

In DBBs and FBBs, 17 saturated fatty acids were found, with palmitic acid (C16:0) being the most abundant, followed by Myristic and Stearic acids. Oleic acid (C18:1n9c) was the most prevalent among the seven identified monounsaturated fatty acids. Polyunsaturated fatty acids predominated, as Linoleic (C18:2n6c) was presented sensibly among the eight identified, and α-Linolenic (C18:3n3) was detected in a considerable amount. Date pits contain high-quality saturated and unsaturated fatty acids such as Lauric, Palmitoleic, Oleic, Linoleic, and Linolenic acids. While oleic acid predominates at 41–58.8%, lauric and linoleic acids in some date species were discovered in 17.8% and 15% of the pit’s oil, respectively [91,92]. Due to their durability and nutritional value, oils high in oleic acid are considered high-quality [92], and their benefits against cardiovascular disease include decreasing total and low-density lipoprotein cholesterol [93]. Date pits may contain such fatty acids, and numerous studies have used them to make functional foods or replace conventional oils [94,95]. In our formula, cow’s samna, walnuts, and sesame seeds were added to both bars to increase the healthier fat content, mainly unsaturated fatty acids, and for taste and satisfaction, as recommended [32]. In addition, adding germinated flax seeds may enhance unsaturated fatty acids, seasoning, and flavor [96].

Regarding phytochemicals and bioactive compounds, FBBs were significantly higher in TPC, TF, and TFL contents and scavenging activity against DPPH and ABTS free radicals than DBBs. The increase in phytochemicals and bioactive compounds may be due to incorporating fruits such as apricot, fig, and raisin, which may possess good phenolic content. Bioactive nutrients in any dietary source can significantly improve their functional qualities [97]. Dates’ bioactive components were strongly associated with antioxidant capacity in various tests [98,99]. Multiple date varietals had 55–75% antioxidant capacity in various investigations [100]. Dates include lots of phytochemicals, including carotenoids and phenolic compounds. Phytochemicals’ antioxidant power boosts food products’ functionality [100]. Date variety, growth or maturation stage, and geographical factors affect phenolic compounds. For instance, polyphenols were high in various date varietals but reduced after date ripening [60]. With so many phenolic components, antioxidant potential can be increased [100].

Color determines acceptability based on food consumers’ visual quality [2,25]. The color of any food product always governs the acceptability level and is a quick identification attribute [22,25]. Both bars showed positive a* values, indicating that DBBs and FBBs possess a reddish color. The b* value for both samples was positive at 27.81 and 28.54 for DBBs and FBBs, respectively. This indicated that DBBs and FBBs showed a yellowish color. Based on a* and b* values, both samples are considered to have an orange hue, with DBBs affected by the lower L* value to make their color brownish. This result is higher in the L* value and lower in the a* value and b* value compared to a date bar [2]. The DBBs are affected by the lower L* value and higher browning index (BI) to make their color brownish. The difference in results may be due to the color of the date content of the DBBs since the date paste is darker than other materials used in the preparation of FBBs [30,38,41,101]. Regarding the total cost of both bars, formulating DBBs compared to FBBs reduced the total cost by 17%, as calculated depending on retail prices.

Sensory evaluation of a new food product provides insights into marketability and consumer acceptability [22,57]. In the present study, sensory evaluation was performed with a 9-point hedonic rating scale, with 1 = dislike extremely and 9 = Like extremely, depending on trained panelists. Results concluded that there were no significant differences between DBBs and FBBs in appearance and odor attributes. This may be due to uniforming the shaping process and adding the same flavoring ingredients as coconut powder, which predominated and caused the main flavor. In contrast, significant differences were found in color, taste, texture, and mouthfeel due to differences in ingredients, as indicated in Table 1. Remarkably, using Sukkari dates instead of fruit mix improved the overall acceptability and encouraged panelists to significantly prefer DBBs over FBBs, as recently indicated [22,38,42].

## 5. Conclusions

Using either Sukkari dates or a fruit mixture as a base, this study aimed to investigate the viability of manufacturing unique high-energy and high-protein bars. Comparisons were made between DBBs and FBBs in terms of their proximate composition, mineral and vitamin content, amino and fatty acid profiles, sugar profiles, phytochemicals, antioxidant activity, and visual color criteria. According to proximate analysis, the DBBs had more ash and crude fiber than the FBBs, while the FBBs had more moisture and fat. The protein content of DBBs and FBBs was similar. Date and fruit content consistently altered the DBB’s and FBB’s sugar profiles. Sucrose was higher in DBBs than FBBs, although fructose, glucose, and maltose were abundant in FBBs. Ca, Cu, Fe, Zn, Mn, and Se were detected in higher amounts in DBBs, while Mg, K, and Na were lower. DBBs had greater scores than the FBBs for the presence of amino acids such as lysine, methionine, histidine, threonine, phenylalanine, and isoleucine. In contrast, the FBB had higher scores for leucine and valine. The most abundant saturated fatty acid in DBBs and FBBs was palmitic acid. Oleic acid was the most common monounsaturated fat. Linoleic acid dominated the eight discovered polyunsaturated fatty acids. High α-linolenic acid concentrations were also found. FBBs had more phytochemicals, bioactive compounds, and free radical scavenging activities than DBBs. In addition, sensory evaluation data showed that panelists significantly preferred DBBs over FBBs. After analyzing and comparing different ingredients used in these bars, Sukkari date is a nutrient-dense, convenient, economical, affordable, and better sugar alternative that helps compensate for calorie content. Therefore, it is strongly advised that dates be used instead of fruits when making high-energy and protein bars. However, the rheological and shelf-life stability of the presented bars should be investigated further.

## Figures and Tables

**Table 1:** Raw ingredients of date-based (DBB) and fruit-based bar (FBB) formulas (g 100 g^−1^).

Ingredients	DBB	FBB
Sukkari date paste	50.0	-
Dried Apricot	-	12.5
Dried fig	-	12.5
Raisin	-	25.0
Milk protein concentrate	10.0	10.0
Whey protein isolate	10.0	10.0
Date syrup	6.5	-
Glucose syrup	-	6.5
Sesame seeds	4.0	4.0
Ground walnuts	5.0	5.0
Whole oat powder	4.0	4.0
Coconut powder	3.0	3.0
Cow’s samna	3.0	3.0
Oat fiber	2.0	2.0
Peanut butter	2.0	2.0
Edible salt	0.5	0.5

DBB: Date-Based Bar, FBB: Fruit-Based Bar.

**Table 2:** Proximate composition of date-based bars (DBB) and fruit-based bars (FBB).

Composition *	DBB	FBB
Moisture	11.22 ± 0.08 ^b^	13.24 ± 0.04 ^a^
Protein	21.4 ± 0.14 ^a^	21.2 ± 0.17 ^a^
Total fat	6.80 ± 0.05 ^b^	7.81 ± 0.02 ^a^
Ash	2.83 ± 0.01 ^a^	2.54 ± 0.01 ^b^
Dietary Fiber	9.00 ± 0.04 ^a^	8.00 ± 0.04 ^b^
Total carbohydrates	57.8 ± 0.06 ^a^	55.24 ± 0.03 ^b^
Energy (kcal)	378 ± 2.72 ^a^	376 ± 1.30 ^a^

*: Presented on 100 g wet weight, ^a,b^: No significant difference (*p* > 0.05) between any two means within the same row with the same superscripted letters; Data are presented as mean ± SE, *n* = 3.

**Table 3:** Content of sugars (g 100 g^−1^) of date-based bars (DBB) and fruit-based bars (FBB).

Sugar Profile	DBB	FBB
Fructose	7.62 ± 0.23 ^b^	12.01 ± 0.52 ^a^
Glucose	7.83 ± 0.32 ^b^	13.53 ± 0.63 ^a^
Sucrose	13.22 ± 0.42 ^a^	<LOQ
Maltose	<LOQ	4.17 ± 0.41 ^a^
Lactose	<LOQ	<LOQ

LOQ: Lower than the detection limit, ^a,b^: No significant difference (*p* > 0.05) between any two means within the same row with the same superscripted letters; Data are presented as mean ± SE, *n* = 3.

**Table 4:** Mineral content (mg Kg^−1^) of date-based bars (DBB) and fruit-based bars (FBB).

Minerals and Trace Elements *	DBB	FBB
Calcium	30.01 ± 0.38 ^a^	5.02 ± 0.03 ^b^
Sodium	25.41 ± 0.02 ^b^	1381.50 ± 1.71 ^a^
Potassium	0.69 ± 0.01 ^b^	492.68 ± 0.88 ^a^
Phosphorus	25.27 ± 0.12 ^a^	25.39 ± 0.23 ^a^
Magnesium	8.03 ± 0.13 ^b^	677.20 ± 0.71 ^a^
Manganese	8.49 ± 0.02 ^a^	0.76 ± 0.01 ^b^
Copper	3.08 ± 0.01 ^a^	0.28 ± 0.01 ^b^
Iron	18.50 ± 0.05 ^a^	2.51 ± 0.01 ^b^
Zinc	21.00 ± 0.06 ^a^	1.90 ± 0.02 ^b^
Selenium	0.11 ± 0.00 ^a^	0.08 ± 0.00 ^b^

*: Presented on wet weight. ^a,b^: No significant difference (*p* > 0.05) between any two means within the same row with the same superscripted letters; Data are presented as mean ± SE, *n* = 3.

**Table 5:** Vitamin content of date-based bars (DBB) and fruit-based bars (FBB).

Vitamins	DBB	FBB
Vitamin A (Retinol), IU 100 g^−1^	53.4 ± 0.12 ^b^	105.2 ± 0.71 ^a^
Vitamin C, mg kg^−1^	Tr	Tr
Vitamin D_3_, IU 100 g^−1^	32.5 ± 0.19 ^a^	Tr
Vitamin E (Tocopherol), mg kg^−1^	15.73 ± 0.03 ^a^	8.22 ± 0.01 ^b^
Vitamin B_l_ (Thiamine), mg kg^−1^	Tr	Tr
Vitamin B_2_ (Riboflavin), mg kg^−1^	0.33 ± 0.01 ^a^	0.30 ± 0.01 ^b^
Vitamin B_3_ (Niacin), mg kg^−1^	Tr	Tr
Vitamin B_5_ (Pantothenic acid), µg kg^−1^	5.70 ± 0.01 ^a^	3.97 ± 0.03 ^b^
Vitamin B_6_ (Pyridoxine), mg kg^−1^	0.25 ± 0.01 ^a^	0.21 ± 0.01 ^b^
Vitamin B_9_ (Folic acid), µg kg^−1^	Tr	45.80 ± 0.21 ^a^
Vitamin B_12_ (Cyanocobalamin), µg kg^−1^	8.12 ± 0.03 ^b^	9.77 ± 0.01 ^a^

Tr: Trace, ^a,b^: No significant difference (*p* > 0.05) between any two means within the same row with the same superscripted letters; Data are presented as mean ± SE, *n* = 3.

**Table 6:** Amino acid composition (g 100 g^−1^ bar) * of date-based bars (DBB) and fruit-based bars (FBB).

	DBB	FBB
Essential Amino acids (EAA)		
Amino Acid		
Leucine	1.37	1.67
Lysine	0.89	0.82
Valine	0.99	1.61
Methionine	0.57	0.38
Histidine	1.15	0.97
Threonine	1.25	0.97
Phenylalanine	1.00	0.72
Isoleucine	0.91	0.88
Cystine	0.14	0.11
Non-Essential Amino acids (NEAA)		
Aspartic Acid	1.58	1.52
Glutamic Acid	5.25	4.95
Serine	1.22	1.42
Glycine	0.60	0.75
Arginine	0.87	0.83
Alanine	0.79	0.71
Tyrosine	0.77	0.75
Proline	0.84	1.29
Essential Amino acids	8.27	8.13
Non-Essential Amino acids	11.92	12.22
EAA/NEAA ratio	0.69	0.67
Total Amino Acids	20.19	20.35

*: Mean of duplicate analysis.

**Table 7:** Amino acids% and calculated biological efficiency, essential amino acid index, estimated protein efficiency ratio, and requirement index of different age groups.

Parameters	DBB	FBB
Total BAAs (mg g^−1^ protein)	136.17	123.76
Total Aromatic AA (mg g^−1^ protein)	82.83	69.44
Total uncharged polar AAs (mg g^−1^ protein)	186.24	188.95
BV	163.00	59.97
EAAI	62.34	59.78
Requirement index (Infants)	133.56	128.06
Requirement index (Preschool child)	145.06	139.09
Requirement index (Schoolchild)	158.73	152.20
Requirement index (Adult)	166.93	160.06

BAAs: Basic amino acids, BV: Calculated biological value, EAAI; Essential amino acid index.

**Table 8:** Fatty acid composition (g 100 g^−1^) of date-based bars (DBB) and fruit-based bars (FBB).

Fatty Acids *	DBB	FBB
Saturated fatty acids		
Fatty Acid	%	%
Butyric (C4:0)	1.14	0.35
Caproic (C6:0)	0.89	0.36
Caprylic (C8:0)	2.38	1.06
Capric (C10:0)	2.29	1.28
Lauric (C12:0)	1.17	6.84
Tridecanoic (C13:0)	0.03	0.02
Myristic (C14:0)	5.79	4.80
Myristoleic (C14:0)	0.26	0.25
Pentadecanoic (C15:0)	0.29	0.28
Palmitic (C16:0)	13.92	13.08
Heptdecanoic (C17:0)	0.12	0.19
Stearic (C18:0)	5.14	4.53
Arachidic (C20:0)	0.24	0.28
Henecosanoic (C21:0)	0.31	0.25
Behenic (C22:0)	0.19	0.24
Tricosanoic (C23:0)	0.05	0
Lignoceric (C24:0)	0.14	0.17
Monounsaturated fatty acids		
Erucic (C22:1n9)	0.03	0.02
Palmitoleic (C16:1)	0.29	0.34
CIS-IOHeptadecanoic (C17:1)	0.07	0.06
C18:1n9t (Elaidic)	0.38	0.56
Oleic (C18:1n9c)	26.25	26.56
Cis-11-Eicosenoic (C20:1n9)	0.28	0.32
Erucic (C22:1n9)	0.02	0.02
Polyunsaturated fatty acid		
Linolelaidic (C18:2n6t)	0.06	0.07
Linoleic (C18:2n6c)	31.7	32.11
γ-Linolenic (C18:3n6)	0.05	0.03
α-Linolenic (C18:3n3)	5.97	5.87
Cis-11,14-Eicosadienoic (C20:2)	0.05	0.02
Cis-8,11,14-Eicosatrienoic (C20:3n6)	0.06	0.01
Cis-11,14,17-Eicosatrienoic (C20:3n3)	0.03	0.02
Cis-13,16-Docosadienoic (C22:2)	0.05	0.04
Total Fatty Acids	99.94	100.00

*: Mean of duplicate analysis.

**Table 9:** Antioxidant activity, total phenolic content, total flavonoids, and total flavonols in date-based bars (DBB) and fruit-based bars (FBB).

Bars	TPC (mg GAE g^−1^)	DPPH-RSA (µmol of TE g^−1^)	ABTS-RSA (µmol of TE g^−1^)	TF (mg QE g^−1^)	TFL (mg QE g^−1^)
DBB	9.12 ± 1.46 ^b^	12.78 ± 0.98 ^a^	18.89 ± 1.09 ^b^	4.99 ± 0.87 ^b^	4.28 ± 0.75 ^b^
FBB	13.74 ± 1.73 ^a^	17.86 ± 1.17 ^a^	23.22 ± 1.85 ^a^	7.98 ± 1.37 ^a^	8.25 ± 0.97 ^a^

^a,b^: No significant difference (*p* > 0.05) between any two means within the same column with the same superscripted letters; Data are presented as mean ± SE, *n* = 3.

**Table 10:** Instrumental and visual color analysis of date-based bars (DBB) and fruit-based bars (FBB).

Treatments	Instrumental Color Parameters						Visual Color
	L*	a*	b*	c	Hᵒ	BI	
DBB	47.58 ± 2.76 ^b^	8.16 ± 0.21 ^a^	27.81 ± 1.03 ^a^	28.98 ± 1.04 ^a^	73.67 ± 0.18 ^b^	96.91 ± 3.05 ^a^	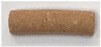
FBB	60.74 ± 1.92 ^a^	4.91 ± 0.06 ^b^	28.54 ± 0.57 ^a^	28.96 ± 0.56 ^a^	80.26 ± 0.30 ^a^	67.60 ± 1.25 ^b^	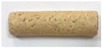

L*, a*, and b* were obtained directly from the Hunter instrument, while C, Hᵒ, and BI were calculated according to the formulated equation in the methods section Equations (1)–(3), ^a,b^: Means that have the same superscript letters within the same columns are not statistically significant (*p* > 0.05), Data are presented as mean ± SE, *n* = 3.

**Table 11:** Sensory evaluation of date-based bars (DBB) and fruit-based bars (FBB), (mean ± SE), *n* = 12.

Treatments	Organoleptical Characteristics						
	Appearance	Color	Taste	Odor	Texture	Mouthfeel	Overall Acceptability
DBB	7.83 ± 0.30 ^a^	7.50 ± 0.23 ^a^	7.42 ± 0.19 ^a^	6.67 ± 0.19 ^a^	8.08 ± 0.26 ^a^	7.42 ± 0.23 ^a^	7.49 ± 0.11 ^a^
FBB	7.58 ± 0.26 ^a^	6.33 ± 0.36 ^b^	6.42 ± 0.26 ^b^	6.83 ± 0.24 ^a^	6.17 ± 0.30 ^b^	6.67 ± 0.28 ^b^	6.67 ± 0.10 ^b^

^a,b^ Means with the same superscript letters within the same columns are not statistically significant (*p* > 0.05).

## Data Availability

The data used to support the findings of this study can be made available by the corresponding author upon request.
